# (*E*)-1,3-Bis(2,3,4,5,6-penta­fluoro­phen­yl)prop-2-en-1-one

**DOI:** 10.1107/S1600536810025675

**Published:** 2010-07-07

**Authors:** Anke Schwarzer, Edwin Weber

**Affiliations:** a Institut für Organische Chemie, TU Bergakademie Freiberg, Leipziger Strasse 29, D-09596 Freiberg/Sachsen, Germany

## Abstract

In the title compound, C_15_H_2_F_10_O, the two perfluorinated arene rings are tilted at an angle of 66.08 (5)° with respect to each other. The olefinic double bond adopts an *E* configuration and the single bond between the olefinic and carbonyl double bonds has an *s*-*trans* conformation. The carbonyl group is not in a coplanar alignment with respect to the neighbouring arene ring (0.963 Å from aryl plane) while being coplanar with regard to the olefinic double bond (0.0805 Å from olefinic bond). The crystal packing does not feature significant hydrogen-bond-type or stacking inter­actions.

## Related literature

For a detailed discussion of fluorinated chalcones, see: Cesarin-Sobrinho & Netto-Ferreira (2002 ▶); Cesarin-Sobrinho *et al.* (2001 ▶). For the crystal structure of the parent chalcone, see: Rabinovich (1970 ▶); Ohkura *et al.* (1973 ▶); Arai *et al.* (1994 ▶); Wu *et al.* (2006 ▶). For a related structure, see: Schwarzer & Weber (2009 ▶). For inter­molecular F⋯F contacts, see: Awwadi *et al.* (2006 ▶). For weak hydrogen bonds, see: Desiraju & Steiner (1999 ▶). For the polymorphism of the non-fluorinated derivative, see: Weygand (1929 ▶).
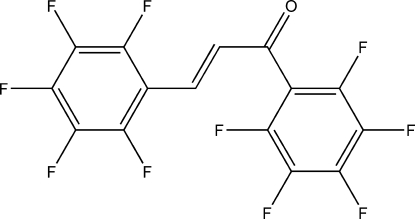


## Experimental

### 

#### Crystal data

C_15_H_2_F_10_O
*M* *_r_* = 388.17
Monoclinic, 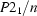

*a* = 11.444 (1) Å
*b* = 9.563 (1) Å
*c* = 12.138 (2) Å
β = 101.414 (3)°
*V* = 1302.1 (3) Å^3^
*Z* = 4
Mo *K*α radiation
μ = 0.22 mm^−1^
*T* = 93 K
0.26 × 0.16 × 0.14 mm

#### Data collection

Bruker SMART CCD area-detector diffractometer
26448 measured reflections
2993 independent reflections
2404 reflections with *I* > 2σ(*I*)
*R*_int_ = 0.037

#### Refinement

*R*[*F* ^2^ > 2σ(*F* ^2^)] = 0.032
*wR*(*F* ^2^) = 0.080
*S* = 1.08
2993 reflections
235 parameters
H-atom parameters constrained
Δρ_max_ = 0.33 e Å^−3^
Δρ_min_ = −0.26 e Å^−3^

### 

Data collection: *SMART* (Bruker, 2007 ▶); cell refinement: *SAINT* (Bruker, 2007 ▶); data reduction: *SAINT*; program(s) used to solve structure: *SHELXS97* (Sheldrick, 2008 ▶); program(s) used to refine structure: *SHELXL97* (Sheldrick, 2008 ▶); molecular graphics: *SHELXTL* (Sheldrick, 2008 ▶); software used to prepare material for publication: *SHELXTL*.

## Supplementary Material

Crystal structure: contains datablocks I, global. DOI: 10.1107/S1600536810025675/im2215sup1.cif

Structure factors: contains datablocks I. DOI: 10.1107/S1600536810025675/im2215Isup2.hkl

Additional supplementary materials:  crystallographic information; 3D view; checkCIF report

## Figures and Tables

**Fig. 1.**
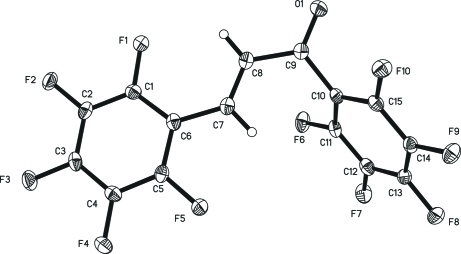
Perspective view of (I), showing 50% probability displacement ellipsoids for the non-H atoms.

## supplementary crystallographic information

### Comment

The title compound (Fig. 1) exhibits a non-planar structure, that can be described by dihedral and torsional angles realting the arene rings and the carbonyl group to each other. The two perfluorinated arene rings are tilted at an angle of 66.08 (5)° with respect to each other. The carbonyl group is tilted with reference to the adjacent perfluoro arene unit showing a torsional angle O1—C9—C10—C15 of 60.3 (2)°. According to Cesarin-Sobrinho *et al.* (2001) this can be expressed as the *s-trans* conformation. The olefinic double bond is fixed in the (*E*)-configuration.

Remarkably, in the crystal structure, the oxygen atom of the polar carbonyl group does not show an intermolecular interaction such as a C—H···O contact (Desiraju & Steiner, 1999). Moreover, neither C—H···F– nor C—H···π contacts and stacking interactions between the perfluorinated arene units occur in the crystal packing. Only closer distances between fluorine atoms and the centre of adjacent perfluorinated aryl rings in the range of 3.0–3.2 Å (150–168°) as well as F···F contacts with distances smaller than the sum of the van-der-Waals-radii can be detected. Nevertheless, there is no indication regarding the angles of the F···F contacts pointing to a *head-on* or *side-on* mode of interaction typical for halogen-halogen contacts (Awwadi *et al.*, 2006).

Drawing a comparison with the parent non-fluorinated compound (*E*)-1,3-diphenyl-2-propen-1-one, which exists in at least four different crystalline polymorphs (Weygand, 1929), a main difference between the decafluorinated chalcone and the nonfluorinated chalcones is the location of the olefinic double bond with reference to the carbonyl double bond. While in the present title compound these bonds are *s-trans*, in the structures of the unfluorinated chalcones they are *s-cis*. The molecular geometries also differ concerning planarity. That is to say, the perfluoro arene units, one with another, show a torsional angle of 66.08 (5)° but for the unfluorinated chalcones the angles are around 12°. Also the torsions involving the adjacent arene and carbonyl groups (2.5, 15.0 and 17.6°) are significantly smaller compared with the fluorinated title compound (60.3 (2)°). Moreover, regarding the packing arrangement, the parent chalcones show a number of intermolecular C—H···O and C—H···π type contacts.

### Experimental

The title compound was obtained from a solution of 2,3,4,5,6-pentafluoroacetophenone and 2,3,4,5,6-pentafluorobenzaldehyde in sulfuric acid. Recrystallization from ethanol yielded 56% single crystals suitable for X-ray crystallography.

### Crystal data

**Table:**

C_15_H_2_F_10_O	*F*(000) = 760
*M**_r_* = 388.17	*D*_x_ = 1.980 Mg m^−^^3^
Monoclinic, *P*2_1_/*n*	Mo *K*α radiation, λ = 0.71073 Å
Hall symbol: -P 2yn	Cell parameters from 6932 reflections
*a* = 11.444 (1) Å	θ = 2.7–31.2°
*b* = 9.563 (1) Å	µ = 0.22 mm^−^^1^
*c* = 12.138 (2) Å	*T* = 93 K
β = 101.414 (3)°	Splitter, yellow
*V* = 1302.1 (3) Å^3^	0.26 × 0.16 × 0.14 mm
*Z* = 4	

### Data collection

**Table:**

Bruker SMART CCD area-detector diffractometer	2404 reflections with *I* > 2σ(*I*)
Radiation source: fine-focus sealed tube	*R*_int_ = 0.037
graphite	θ_max_ = 27.5°, θ_min_ = 2.2°
phi and ω scans	*h* = −14→14
26448 measured reflections	*k* = −12→11
2993 independent reflections	*l* = −15→15

### Refinement

**Table:**

Refinement on *F*^2^	Primary atom site location: structure-invariant direct methods
Least-squares matrix: full	Secondary atom site location: difference Fourier map
*R*[*F*^2^ > 2σ(*F*^2^)] = 0.032	Hydrogen site location: inferred from neighbouring sites
*wR*(*F*^2^) = 0.080	H-atom parameters constrained
*S* = 1.08	*w* = 1/[σ^2^(*F*_o_^2^) + (0.0302*P*)^2^ + 0.8152*P*] where *P* = (*F*_o_^2^ + 2*F*_c_^2^)/3
2993 reflections	(Δ/σ)_max_ = 0.001
235 parameters	Δρ_max_ = 0.33 e Å^−^^3^
0 restraints	Δρ_min_ = −0.25 e Å^−^^3^

### Special details

**Table:**

Geometry. All e.s.d.'s (except the e.s.d. in the dihedral angle between two l.s. planes) are estimated using the full covariance matrix. The cell e.s.d.'s are taken into account individually in the estimation of e.s.d.'s in distances, angles and torsion angles; correlations between e.s.d.'s in cell parameters are only used when they are defined by crystal symmetry. An approximate (isotropic) treatment of cell e.s.d.'s is used for estimating e.s.d.'s involving l.s. planes.
Refinement. Refinement of *F*^2^ against ALL reflections. The weighted *R*-factor *wR* and goodness of fit *S* are based on *F*^2^, conventional *R*-factors *R* are based on *F*, with *F* set to zero for negative *F*^2^. The threshold expression of *F*^2^ > σ(*F*^2^) is used only for calculating *R*-factors(gt) *etc*. and is not relevant to the choice of reflections for refinement. *R*-factors based on *F*^2^ are statistically about twice as large as those based on *F*, and *R*- factors based on ALL data will be even larger.

### Fractional atomic coordinates and isotropic or equivalent isotropic displacement parameters (Å^2^)

**Table:**

	*x*	*y*	*z*	*U*_iso_*/*U*_eq_	
O1	−0.08561 (10)	0.88461 (13)	0.74475 (9)	0.0217 (3)	
F1	0.11079 (8)	0.82449 (11)	1.13198 (7)	0.0219 (2)	
F2	0.25675 (9)	0.81099 (10)	1.33222 (7)	0.0229 (2)	
F3	0.47708 (9)	0.92977 (11)	1.36477 (8)	0.0252 (2)	
F4	0.54620 (8)	1.07257 (10)	1.19539 (8)	0.0235 (2)	
F5	0.39739 (8)	1.09616 (10)	0.99452 (8)	0.0214 (2)	
F6	0.21736 (9)	0.74714 (11)	0.73250 (8)	0.0252 (2)	
F7	0.34571 (9)	0.82160 (11)	0.57810 (8)	0.0264 (2)	
F8	0.29589 (9)	1.06719 (11)	0.46224 (8)	0.0237 (2)	
F9	0.10919 (9)	1.22599 (10)	0.49466 (8)	0.0228 (2)	
F10	−0.02162 (8)	1.14862 (10)	0.64486 (8)	0.0217 (2)	
C1	0.21650 (14)	0.89021 (17)	1.14490 (13)	0.0170 (3)	
C2	0.29072 (15)	0.88141 (17)	1.24884 (13)	0.0184 (3)	
C3	0.40208 (15)	0.94296 (17)	1.26555 (13)	0.0187 (3)	
C4	0.43704 (14)	1.01588 (17)	1.17940 (13)	0.0182 (3)	
C5	0.36026 (14)	1.02514 (17)	1.07695 (12)	0.0167 (3)	
C6	0.24865 (14)	0.96066 (17)	1.05466 (12)	0.0163 (3)	
C7	0.18133 (14)	0.95916 (17)	0.93890 (13)	0.0170 (3)	
H7	0.2177	1.0056	0.8852	0.020*	
C8	0.07515 (15)	0.90086 (17)	0.89895 (13)	0.0189 (3)	
H8	0.0346	0.8541	0.9493	0.023*	
C9	0.01954 (14)	0.90743 (17)	0.77860 (13)	0.0168 (3)	
C10	0.09586 (14)	0.94777 (17)	0.69495 (12)	0.0160 (3)	
C11	0.18995 (15)	0.86724 (17)	0.67481 (13)	0.0180 (3)	
C12	0.25647 (14)	0.90341 (18)	0.59587 (13)	0.0193 (3)	
C13	0.23042 (14)	1.02638 (18)	0.53571 (12)	0.0181 (3)	
C14	0.13576 (14)	1.10771 (16)	0.55273 (13)	0.0165 (3)	
C15	0.06936 (14)	1.06744 (17)	0.63041 (13)	0.0167 (3)	

### Atomic displacement parameters (Å^2^)

**Table:**

	*U*^11^	*U*^22^	*U*^33^	*U*^12^	*U*^13^	*U*^23^
O1	0.0186 (6)	0.0239 (6)	0.0216 (6)	−0.0039 (5)	0.0013 (5)	−0.0006 (5)
F1	0.0197 (5)	0.0284 (6)	0.0177 (5)	−0.0028 (4)	0.0041 (4)	0.0009 (4)
F2	0.0242 (5)	0.0301 (6)	0.0156 (4)	0.0031 (4)	0.0067 (4)	0.0050 (4)
F3	0.0228 (5)	0.0339 (6)	0.0163 (5)	0.0038 (4)	−0.0022 (4)	0.0021 (4)
F4	0.0190 (5)	0.0273 (6)	0.0224 (5)	−0.0040 (4)	−0.0009 (4)	−0.0008 (4)
F5	0.0223 (5)	0.0232 (5)	0.0183 (5)	−0.0049 (4)	0.0031 (4)	0.0029 (4)
F6	0.0330 (6)	0.0224 (5)	0.0201 (5)	0.0108 (4)	0.0050 (4)	0.0059 (4)
F7	0.0242 (5)	0.0338 (6)	0.0216 (5)	0.0121 (5)	0.0058 (4)	−0.0012 (4)
F8	0.0231 (5)	0.0312 (6)	0.0186 (5)	−0.0020 (4)	0.0086 (4)	−0.0002 (4)
F9	0.0290 (5)	0.0168 (5)	0.0237 (5)	0.0005 (4)	0.0077 (4)	0.0054 (4)
F10	0.0203 (5)	0.0197 (5)	0.0265 (5)	0.0047 (4)	0.0081 (4)	0.0026 (4)
C1	0.0154 (8)	0.0173 (8)	0.0190 (8)	0.0018 (6)	0.0049 (6)	−0.0028 (6)
C2	0.0226 (8)	0.0192 (8)	0.0149 (7)	0.0050 (7)	0.0072 (6)	0.0017 (6)
C3	0.0208 (8)	0.0206 (8)	0.0133 (7)	0.0069 (7)	−0.0001 (6)	−0.0016 (6)
C4	0.0156 (8)	0.0178 (8)	0.0204 (8)	0.0008 (6)	0.0020 (6)	−0.0035 (6)
C5	0.0218 (8)	0.0146 (8)	0.0139 (7)	0.0018 (6)	0.0045 (6)	−0.0002 (6)
C6	0.0183 (8)	0.0156 (8)	0.0149 (7)	0.0038 (6)	0.0029 (6)	−0.0027 (6)
C7	0.0208 (8)	0.0152 (8)	0.0153 (7)	0.0031 (6)	0.0042 (6)	−0.0004 (6)
C8	0.0225 (8)	0.0189 (8)	0.0154 (7)	0.0002 (7)	0.0043 (6)	0.0002 (6)
C9	0.0197 (8)	0.0141 (8)	0.0165 (7)	0.0000 (6)	0.0032 (6)	−0.0006 (6)
C10	0.0160 (8)	0.0183 (8)	0.0123 (7)	−0.0021 (6)	−0.0008 (6)	−0.0025 (6)
C11	0.0215 (8)	0.0170 (8)	0.0136 (7)	0.0020 (6)	−0.0013 (6)	0.0009 (6)
C12	0.0182 (8)	0.0231 (9)	0.0156 (7)	0.0043 (7)	0.0009 (6)	−0.0059 (6)
C13	0.0185 (8)	0.0235 (9)	0.0122 (7)	−0.0035 (7)	0.0028 (6)	−0.0031 (6)
C14	0.0187 (8)	0.0148 (8)	0.0146 (7)	−0.0028 (6)	−0.0003 (6)	−0.0005 (6)
C15	0.0161 (8)	0.0164 (8)	0.0162 (7)	−0.0010 (6)	−0.0002 (6)	−0.0034 (6)

### Geometric parameters (Å, °)

**Table:**

O1—C9	1.212 (2)	C4—C5	1.376 (2)
F1—C1	1.3446 (19)	C5—C6	1.396 (2)
F2—C2	1.3358 (18)	C6—C7	1.463 (2)
F3—C3	1.3393 (17)	C7—C8	1.338 (2)
F4—C4	1.3401 (19)	C7—H7	0.9500
F5—C5	1.3458 (18)	C8—C9	1.475 (2)
F6—C11	1.3495 (19)	C8—H8	0.9500
F7—C12	1.3375 (19)	C9—C10	1.514 (2)
F8—C13	1.3317 (18)	C10—C11	1.384 (2)
F9—C14	1.3356 (18)	C10—C15	1.386 (2)
F10—C15	1.3378 (19)	C11—C12	1.381 (2)
C1—C2	1.377 (2)	C12—C13	1.385 (2)
C1—C6	1.396 (2)	C13—C14	1.382 (2)
C2—C3	1.382 (2)	C14—C15	1.378 (2)
C3—C4	1.380 (2)		
			
F1—C1—C2	117.12 (14)	C9—C8—H8	119.2
F1—C1—C6	120.32 (14)	O1—C9—C8	122.21 (15)
C2—C1—C6	122.54 (15)	O1—C9—C10	118.96 (14)
F2—C2—C1	120.38 (15)	C8—C9—C10	118.81 (14)
F2—C2—C3	120.11 (14)	C11—C10—C15	116.77 (15)
C1—C2—C3	119.48 (15)	C11—C10—C9	123.27 (14)
F3—C3—C4	119.90 (15)	C15—C10—C9	119.90 (14)
F3—C3—C2	120.01 (14)	F6—C11—C12	118.02 (14)
C4—C3—C2	120.08 (14)	F6—C11—C10	119.40 (14)
F4—C4—C5	121.00 (15)	C12—C11—C10	122.56 (15)
F4—C4—C3	119.75 (14)	F7—C12—C11	120.59 (15)
C5—C4—C3	119.21 (15)	F7—C12—C13	120.21 (15)
F5—C5—C4	117.72 (14)	C11—C12—C13	119.20 (15)
F5—C5—C6	119.36 (13)	F8—C13—C14	119.78 (15)
C4—C5—C6	122.88 (15)	F8—C13—C12	120.72 (15)
C5—C6—C1	115.74 (14)	C14—C13—C12	119.50 (15)
C5—C6—C7	118.58 (14)	F9—C14—C15	119.94 (14)
C1—C6—C7	125.35 (15)	F9—C14—C13	120.08 (14)
C8—C7—C6	128.34 (15)	C15—C14—C13	119.98 (15)
C8—C7—H7	115.8	F10—C15—C14	118.45 (14)
C6—C7—H7	115.8	F10—C15—C10	119.60 (14)
C7—C8—C9	121.62 (15)	C14—C15—C10	121.93 (15)
C7—C8—H8	119.2		
			
F1—C1—C2—F2	−0.5 (2)	O1—C9—C10—C11	−116.74 (18)
C6—C1—C2—F2	−178.97 (14)	C8—C9—C10—C11	64.8 (2)
F1—C1—C2—C3	177.77 (14)	O1—C9—C10—C15	60.3 (2)
C6—C1—C2—C3	−0.7 (2)	C8—C9—C10—C15	−118.11 (17)
F2—C2—C3—F3	1.3 (2)	C15—C10—C11—F6	−177.84 (14)
C1—C2—C3—F3	−177.02 (14)	C9—C10—C11—F6	−0.7 (2)
F2—C2—C3—C4	−179.91 (15)	C15—C10—C11—C12	0.9 (2)
C1—C2—C3—C4	1.8 (2)	C9—C10—C11—C12	178.04 (15)
F3—C3—C4—F4	0.3 (2)	F6—C11—C12—F7	−0.4 (2)
C2—C3—C4—F4	−178.47 (14)	C10—C11—C12—F7	−179.14 (14)
F3—C3—C4—C5	178.17 (14)	F6—C11—C12—C13	−179.81 (14)
C2—C3—C4—C5	−0.6 (2)	C10—C11—C12—C13	1.4 (2)
F4—C4—C5—F5	−1.7 (2)	F7—C12—C13—F8	−2.2 (2)
C3—C4—C5—F5	−179.55 (14)	C11—C12—C13—F8	177.26 (14)
F4—C4—C5—C6	176.10 (14)	F7—C12—C13—C14	178.14 (14)
C3—C4—C5—C6	−1.7 (2)	C11—C12—C13—C14	−2.4 (2)
F5—C5—C6—C1	−179.47 (14)	F8—C13—C14—F9	0.8 (2)
C4—C5—C6—C1	2.7 (2)	C12—C13—C14—F9	−179.51 (14)
F5—C5—C6—C7	6.8 (2)	F8—C13—C14—C15	−178.60 (13)
C4—C5—C6—C7	−171.00 (15)	C12—C13—C14—C15	1.1 (2)
F1—C1—C6—C5	−179.90 (14)	F9—C14—C15—F10	0.5 (2)
C2—C1—C6—C5	−1.5 (2)	C13—C14—C15—F10	179.96 (13)
F1—C1—C6—C7	−6.7 (2)	F9—C14—C15—C10	−178.06 (13)
C2—C1—C6—C7	171.73 (15)	C13—C14—C15—C10	1.4 (2)
C5—C6—C7—C8	178.28 (16)	C11—C10—C15—F10	179.11 (13)
C1—C6—C7—C8	5.2 (3)	C9—C10—C15—F10	1.9 (2)
C6—C7—C8—C9	−179.30 (15)	C11—C10—C15—C14	−2.3 (2)
C7—C8—C9—O1	−162.80 (16)	C9—C10—C15—C14	−179.54 (14)
C7—C8—C9—C10	15.6 (2)		
